# Analysis of a poly(ε-decalactone)/silver nanowire composite as an electrically conducting neural interface biomaterial

**DOI:** 10.1186/s42490-019-0010-3

**Published:** 2019-04-15

**Authors:** Katarzyna Krukiewicz, Jorge Fernandez, Małgorzata Skorupa, Daria Więcławska, Anup Poudel, Jose-Ramon Sarasua, Leo R. Quinlan, Manus J. P. Biggs

**Affiliations:** 1 Centre for Research in Medical Devices (CURAM), Galway Biosciences Research Building, 118 Corrib Village, Newcastle, Galway, Ireland; 2 Department of Physical Chemistry and Technology of Polymers, Silesian University of Technology, M.Strzody 9, 44-100 Gliwice, Poland; 3 Polimerbio, S.L, Paseo Mikeletegi 83, 20009 Donostia-San Sebastian, Spain; 4 Department of Mining-Metallurgy Engineering and Materials Science, POLYMAT, University of the Basque Country (UPV/EHU), School of Engineering, Alameda de Urquijo s/n, 48013 Bilbao, Spain; 5 Department of Physiology, National University of Ireland, Galway, University Road, Galway, Ireland

**Keywords:** Polyesters, Poly(ε-decalactone), Silver nanowires, Neural interfaces, Neural stimulation

## Abstract

**Background:**

Advancement in polymer technologies, facilitated predominantly through chemical engineering approaches or through the identification and utilization of novel renewable resources, has been a steady focus of biomaterials research for the past 50 years. Aliphatic polyesters have been exploited in numerous biomedical applications including the formulation of soft-tissue sutures, bone fixation devices, cardiovascular stents etc. Biomimetic ‘soft’ polymer formulations are of interest in the design of biological interfaces and specifically, in the development of implantable neuroelectrode systems intended to interface with neural tissues. Critically, soft polymer formulations have been shown to address the challenges associated with the disregulation of mechanotransductive processes and micro-motion induced inflammation at the electrode/tissue interface. In this study, a polyester-based poly(ε-decalactone)/silver nanowire (EDL:Ag) composite was investigated as a novel electrically active biomaterial with neural applications.

Neural interfaces were formulated through spin coating of a polymer/nanowire formulation onto the surface of a Pt electrode to form a biocompatible EDL matrix supported by a percolated network of silver nanowires. As-formed EDL:Ag composites were characterized by means of infrared spectroscopy, scanning electron microscopy and electrochemical methods, with their cytocompatibility assessed using primary cultures of a mixed neural population obtained from the ventral mesencephalon of Sprague-Dawley rat embryos.

**Results:**

Electrochemical characterization of various EDL:Ag composites indicated EDL:Ag 10:1 as the most favourable formulation, exhibiting high charge storage capacity (8.7 ± 1.0 mC/cm^2^), charge injection capacity (84.3 ± 1.4 μC/cm^2^) and low impedance at 1 kHz (194 ± 28 Ω), outperforming both pristine EDL and bare Pt electrodes. The in vitro biological evaluation showed that EDL:Ag supported significant neuron viability in culture and to promote neurite outgrowth, which had the average length of 2300 ± 6 μm following 14 days in culture, 60% longer than pristine EDL and 120% longer than bare Pt control substrates.

**Conclusions:**

EDL:Ag nanocomposites are shown to serve as robust neural interface materials, possessing favourable electrochemical characteristics together with high neural cytocompatibility.

## Background

Aliphatic polyesters are among the most widely applied polymeric materials in biomedical engineering [1]. Thanks to their suitable physicochemical properties and ease of fabrication, polyesters have been successfully used as soft-tissue sutures, cardiovascular stents and bone fixation devices [2, 3], as well as advanced drug delivery systems [4, 5]. Additional advantages of polyesters in biomedical engineering stem from their biodegradable and bioresorbable properties which facilitate their use as temporary drug carriers or implants [6]. The most commonly used polyesters, i.e. polylactic acid, poly(lactic-co-glycolic acid), poly(ε-caprolactone) and poly-3-hydroxybutyrate have been exploited in biomedical engineering over the last 50 years, with the first polyester-based medical device being approved by the Food and Drug Administration in 1969 [1]. Since then, much research has been focused on the improvement of polyester technology, mainly through chemical and physical modification of existing polymer formulations [2] or through the identification and utilization of novel renewable sources [7].

ε-Decalactone (εDL) belongs to the class of lactones composed of 10 carbon atoms, which can be polymerized to form a linear polyester [8, 9]. εDL is a commercially available, renewable material produced through fungal technology, and is commonly used in the flavouring and fragrance industries [9]. The structural similarity of εDL with ε-caprolactone indicates this polymer to be a promising candidate for numerous biomedical applications. Consequently, the synthesis and characteristics of several copolymers derived from ε-decalactone have been recently described [8]. Interestingly, incorporation of εDL into these copolymer formulations was shown to significantly decrease the polymer stiffness while not impacting on the material’s mechanical strength. Moreover, the presence of εDL-derived domains hinders the hydrolysis of the copolymer due to a steric effect which results in a mechanically soft and slowly degradable material with assumed biocompatibility.

Brain machine interfaces have shown great promises as a way to treat central nervous system disorders such as deafness, paralysis, epilepsy and Parkinson’s disease [10], already reaching notable clinical successes [11]. To facilitate integration with surrounding tissue, neural implants should possess chemical and physical properties analogous to the properties typical of the neural microenvironment, including wettability, roughness and mechanical rigidity [12]. Biomimetic ‘soft’ polymer formulations are of interest in the design of biological interfaces and specifically, in the development of implantable neuroelectrode systems intended to interface with neural tissues. In particular, mechanically soft formulations have been shown to address the challenges associated with the disregulation of mechanotransductive processes [13] and micro-motion induced inflammation at the electrode/tissue interface [14]. Critically, peri-implant gliosis has been shown to significantly reduce chronic neuroelectrode functionality in vivo [15]. It follows that metal electrodes can be functionalized with biocompatible organic coatings with an aim to provide a biointerface with tailorable electromechanical properties that promote electrode integration as well as chronic functionality [16, 17]. Because the functional coating must be both biocompatible and also electrically conducting, the range of biomaterials that can be used in this specific application is limited to either intrinsically conducting polymers, such as polypyrrole [18] or poly(3,4-ethylenedioxythiophene) [16] or composite materials formed from a conducting filler (carbon nanotubes, metal particles, etc) and biocompatible polymer matrix [19].

Here, poly(ε-decalactone) (EDL) is explored as a mechanically soft and biocompatible polymeric matrix reinforced with highly conductive silver nanowires (AgNWs). Thanks to their advantageous physical properties, including excellent electrical and optical properties, as well as mechanical robustness, AgNWs have recently received a great attention as a novel electrode material [20], being successfully used to fabricate transparent and flexible electrodes [21], highly conductive microelectrodes or biocompatible hydrogels [20], as well as stretchable fibers for optoelectronic probing of spinal cord circuits [22]. In this work, functionalised Pt macro-electrodes presenting a functional EDL:Ag composite coating were formed through a spin coating process and were characterized by means of FTIR and SEM. By changing the concentration of filler, the percolation threshold, i.e. the minimum amount of AgNWs required for the material to be conductive, was determined based on the electrochemical performance of the composites. To assess the cytocompatibility of the coatings, mixed neural populations obtained from the mesencephalon of embryonic Sprague–Dawley rats, (the midbrain is a target of deep brain stimulation [23]), were cultured on the optimal EDL:Ag formulation and cellular proliferation, morphology and neural depolarisation in response to electrical stimulation were assessed in vitro.

## Results

### Rheological testing

**Fig. 1:**
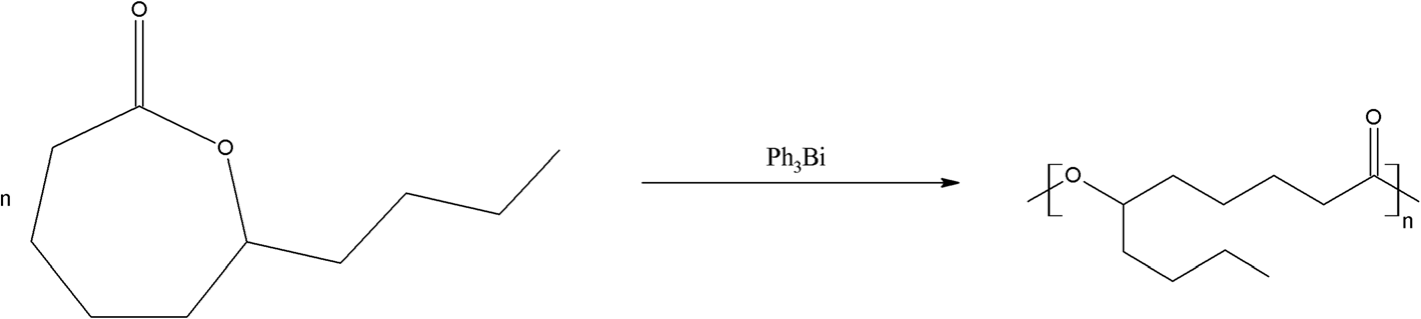
Ring opening polymerization of εDL to form EDL. Schematic representation of the polymerization reaction of ε-decalactone (εDL) with Ph_3_Bi as the catalyst to form poly(ε-decalactone) (EDL)

**Fig. 2:**
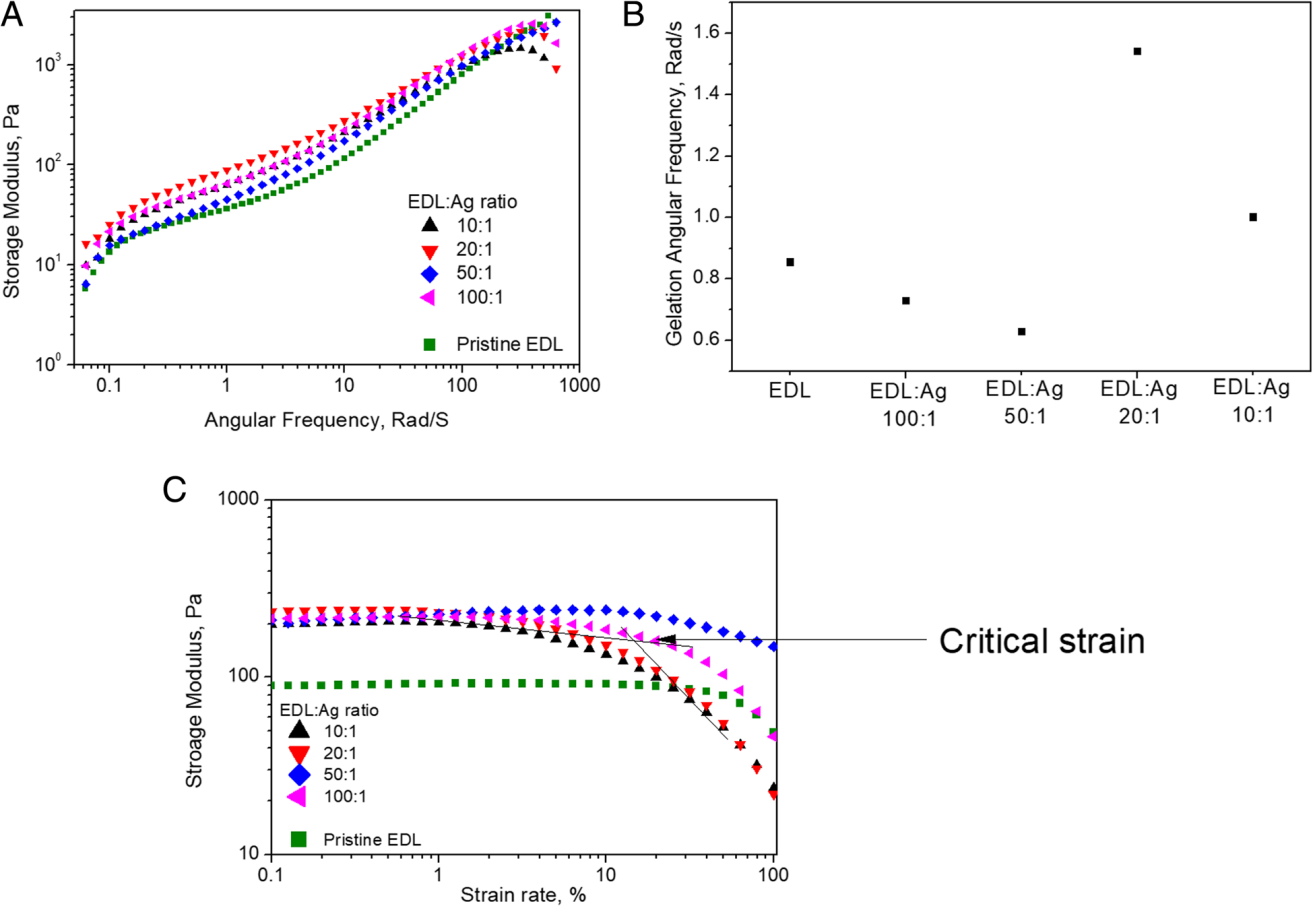
Rheological behaviour of pristine EDL and EDL:Ag nanocomposite formulations. The storage modulus over a range of frequencies under a constant strain rate of 1% (**a**). The gelation angular frequency under a constant strain of 1% (**b**) and storage modulus over a range of strain rates under constant angular frequency of 6.24 rad/sec (**c**)

The ring-opening polymerization process of ε-decalactone is shown in Fig. 1. The as-formed poly(ε-decalactone) (EDL) presented an average molecular weight of 86.6 kg mol^− 1^ and a dispersity of 2.22 and was fully amorphous with a glass transition temperature at − 50.2 °C. At room temperature, EDL was observed to act as a viscous liquid. To formulate an electrically conducting composite material, pristine EDL was mixed with a AgNWs ethanol dispersion (20 mg/ml) in different mass ratios, from 100:1 to 10:1. Figure 2a-c shows the storage modulus over the range of frequencies under constant strain rate of 1%, gelation angular frequency under constant strain of 1% and storage modulus over a range of strain rates under constant angular frequency at 6.24 rad/sec. A very slight change in gelation angular frequency (the frequency at which a material becomes a gel with a loss tangent value equal to 1) was observed with composites possessing a low percentage of Ag (up to EDL:Ag 50:1). Higher concentrations of AgNWs resulted in a higher gelation frequency suggesting elastic behavior even at higher frequency, however, this change was very small (0.8 rad/sec). Similarly, with frequencies below 0.1 rad/sec, the storage modulus was observed to remain below 30 Pa (Fig. 2a). The highest storage modulus was observed in the EDL:Ag 20:1 formulation suggesting enhanced elastic behavior in this material. This result could also be observed by the higher gelation frequency in EDL:Ag 20:1 nanocomposites (Fig. 2b). However, when the ratio of EDL:Ag was decreased to 10:1, the storage modulus as well as gelation frequency was also observed to decrease. Below a strain rate of 10%, the storage modulus demonstrated a modest increase (ca. 10%) upon addition of AgNWs. As depicted in Fig. 2c, the critical strain of the EDL was changed by the addition of AgNWs, and below 10% strain, the structure of all material formulations remained intact. The lowest critical strain rate was observed in the composite formulation with an EDL to Ag ratio of 10:1.

### Surface characterization

**Fig. 3:**
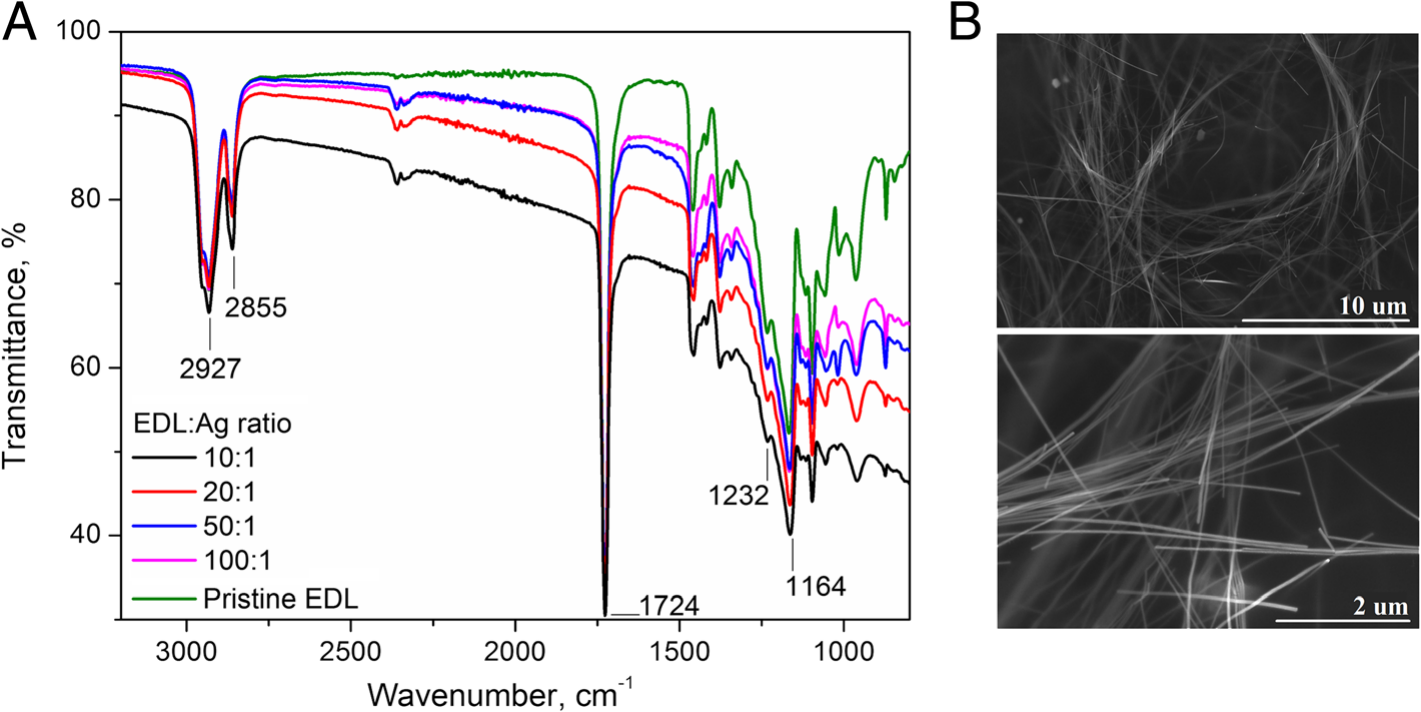
FTIR and SEM analysis of pristine EDL and EDL:Ag nanocomposites. FTIR spectra of pristine EDL and EDL:Ag composites (**a**); representative SEM images of a EDL:Ag 10:1 composite showing a network of AgNWs within an EDL matrix (**b**)

To formulate an electrically conducting coating, EDL:Ag formulations were spin coated onto a Pt-covered glass slide. Different concentrations of EDL (from 5 to 30%) were used to optimize the coating process. It was observed that higher concentrations (> 15%) resulted in the formation of a non-uniform layer, and lower concentrations (< 10%) resulted in the formation of non-continuous films with regions of exposed bare platinum. A 10% *w*/*v* solution of EDL in tetrahydrofuran was found to give the most homogeneous coating with the uniform thickness of 5.8 ± 0.8 μm. FTIR spectra of the resulting polymer/metal composites (Fig. 3a) demonstrated distinctive bands attributable to EDL, namely the absorption band assigned to the C–H hydroxyl groups asymmetric stretching (2927 cm^− 1^), the band assigned to C-H hydroxyl groups symmetric stretching (2855 cm^− 1^), the absorption band assigned to -C=O stretching vibrations of the ester carbonyl group (1724 cm^− 1^) [24], as well as the bands assigned to C-O-C asymmetric stretching (1232 cm^− 1^) and C-O-C symmetric stretching (1164 cm^− 1^), as reported previously with commercial polycaprolactone [25]. Figure 3b shows the SEM images of a representative EDL:Ag 10:1 composite. It could be observed that a high degree of dispersion was achieved with no apparent bundles of AgNWs, which instead formed coiled and interconnected woven structures.

### Electrochemical behaviour and charge storage capacity

**Fig. 4:**
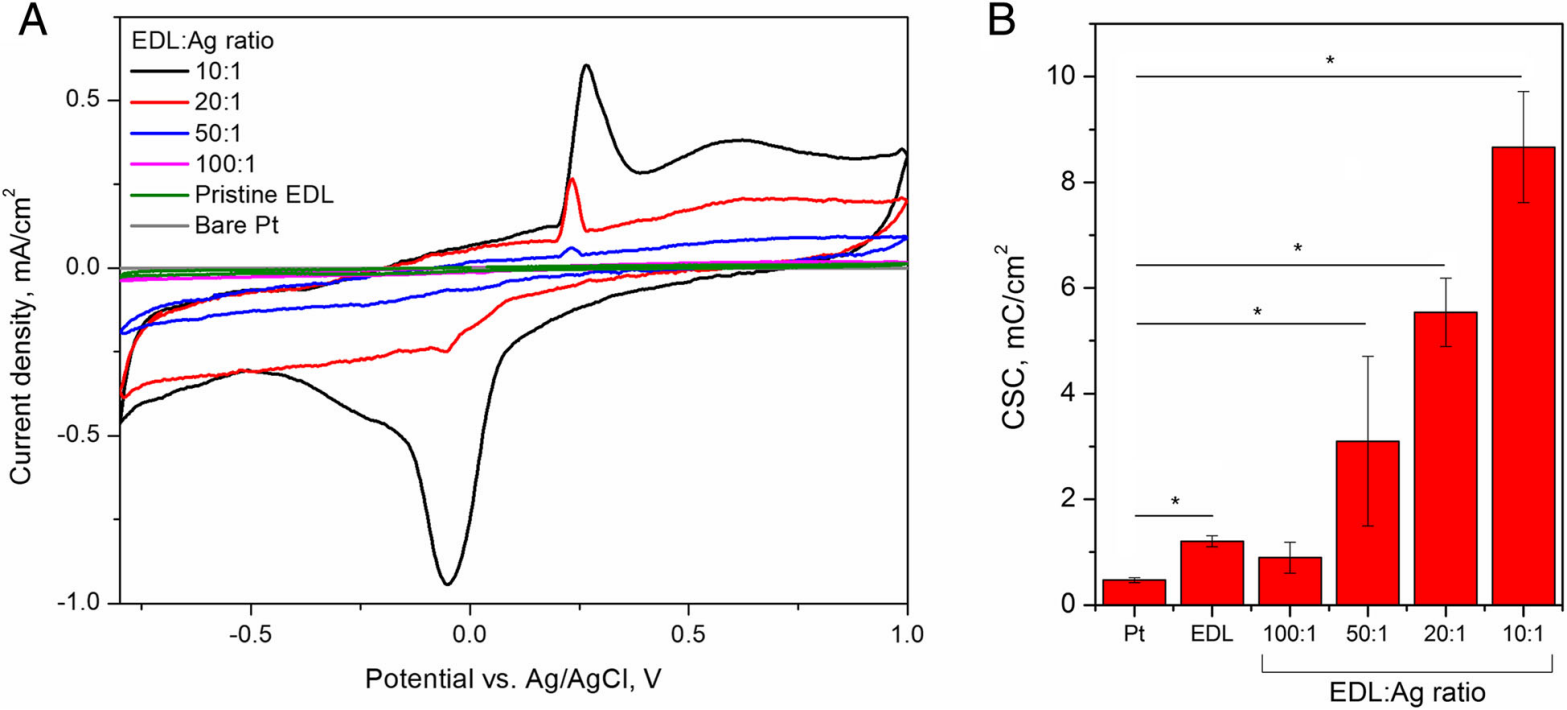
Electrochemical behaviour and charge storage capacity of pristine EDL and EDL:Ag nanocomposites. Cyclic voltammetric curves (**a**) and charge storage capacity (CSC) values (**b**) of Pt electrodes coated with EDL:Ag composites as well as control pristine EDL coated and bare Pt electrodes, indicating the electroactivity of AgNWs and their role in increasing the CSC of EDL:Ag composite coatings;  = *p* < 0.05, *N* = 3

The cyclic voltammetric (CV) curves of EDL:Ag composites (Fig. 4a) showed a distinctive reduction-oxidation system at the potentials of − 0.1 V (reduction) and 0.25 V (oxidation). The increase in current densities noted for the increasing mass ratio of AgNWs in the composite resulted in a significant increase in the charge storage capacity (CSC) (Fig. 4b). Consequently, the CSC reached a maximum value of 8.7 ± 1.0 mC/cm^2^ for EDL:Ag 10:1, which was almost 20 times higher than that of bare Pt substrates (0.5 ± 0.1 mC/cm^2^) and 7 times higher than that of pristine EDL coated Pt electrodes (1.2 ± 0.2 mC/cm^2^).

### Charge injection capacity

**Fig. 5:**
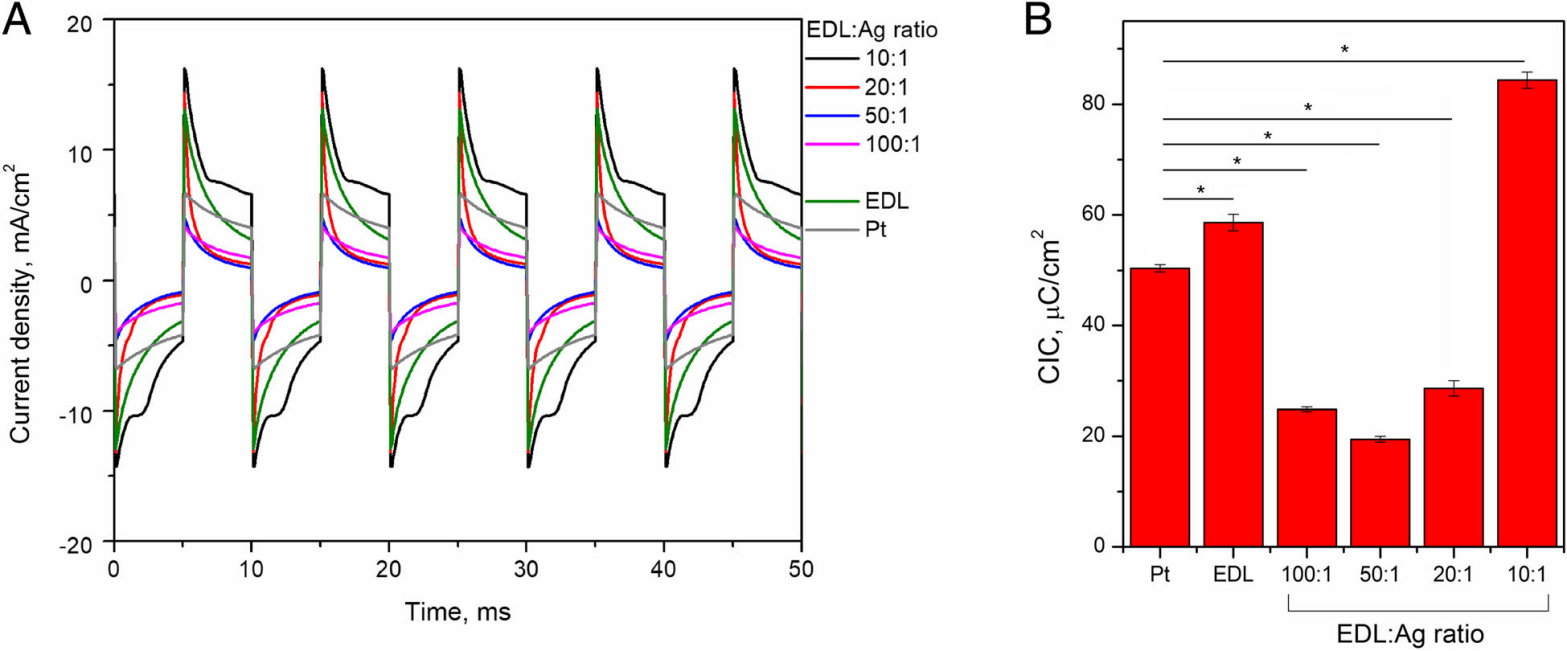
Charge injection capacity analysis of pristine EDL and EDL:Ag nanocomposites. Chronoamperometric curves (**a**) and charge injection capacity (CIC) values (**b**) of EDL:Ag composites as well as pristine EDL and bare Pt electrodes, indicating the effect of AgNW content on the nanocomposite CIC;  = *p* < 0.05, *N* = 3

To simulate the conditions of electrical stimulation of neural tissue [26–31], a cathodic-first biphasic potential stimulation protocol was used in which potentials of − 0.5 / 0.5 V were applied for 5 ms. The current responses of Pt electrodes coated with EDL:Ag composites and Pt electrodes coated with pristine EDL and bare Pt electrodes were presented in the form of chronoamperometric curves (Fig. 5a). It was observed that Pt electrodes coated with an EDL:Ag 10:1 formulation demonstrated the highest electrical currents coupled with a stable signal relative to other experimental and control electrodes. By integrating the current density with respect to time, the charge density achievable during electrical stimulation was determined and expressed as charge injection capacity (CIC) (Fig. 5b). EDL:Ag 10:1 coated electrodes possessed the CIC of 84.3 ± 1.4 μC/cm^2^, pristine EDL possessed the CIC of 58.6 ± 1.5 μC/cm^2^ and bare Pt possessed the CIC of 50.3 ± 0.7 μC/cm^2^.

### Electrochemical impedance characteristics

**Fig. 6:**
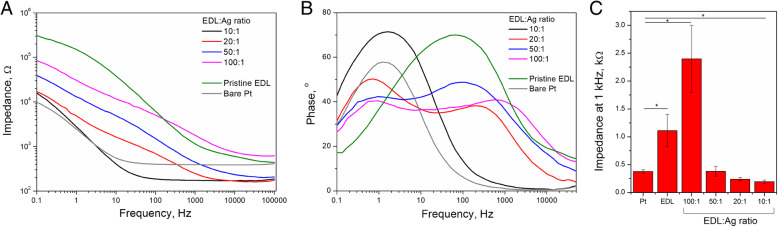
Impedance analysis of pristine EDL and EDL:Ag nanocomposites. Electrochemical impedance spectroscopy data in the form of Bode plots showing the frequency-dependent behaviour of the impedance modulus (**a**) and phase angle (**b**) of EDL:Ag composites as well as pristine EDL coated and bare Pt electrodes; impedances at 1 kHz (**c**) indicate the role of AgNWs in reducing the electrical impedance of EDL matrix;  = *p* < 0.05, *N* = 3

Electrochemical impedance data in the form of a Bode plot for EDL:Ag composites as well as pristine EDL and bare Pt substrates (Fig. 6a) indicated that the impedance modulus as a function of frequency was lowest for Pt electrodes (for frequencies below 10 Hz) and EDL:Ag 10:1 (for frequencies above 10 Hz). Upon analysis of the phase angle profile (Fig. 6b) of pristine EDL relative to bare Pt, a significant shift in the frequency peak was observed (1 and 100 Hz for bare Pt and pristine EDL, respectively). The EDL:Ag 10:1 formulation was also found to have the lowest impedance at 1 kHz (Fig. 6c). The impedance modulus of EDL:Ag 10:1 (194 ± 28 Ω) at 1 kHz was reduced by a factor of two relative to bare Pt electrodes (378 ± 32 Ω) and a factor of five relative to pristine EDL coated electrodes (1111 ± 601 Ω).

### Cytocompatibility and neural stimulation studies

**Fig. 7:**
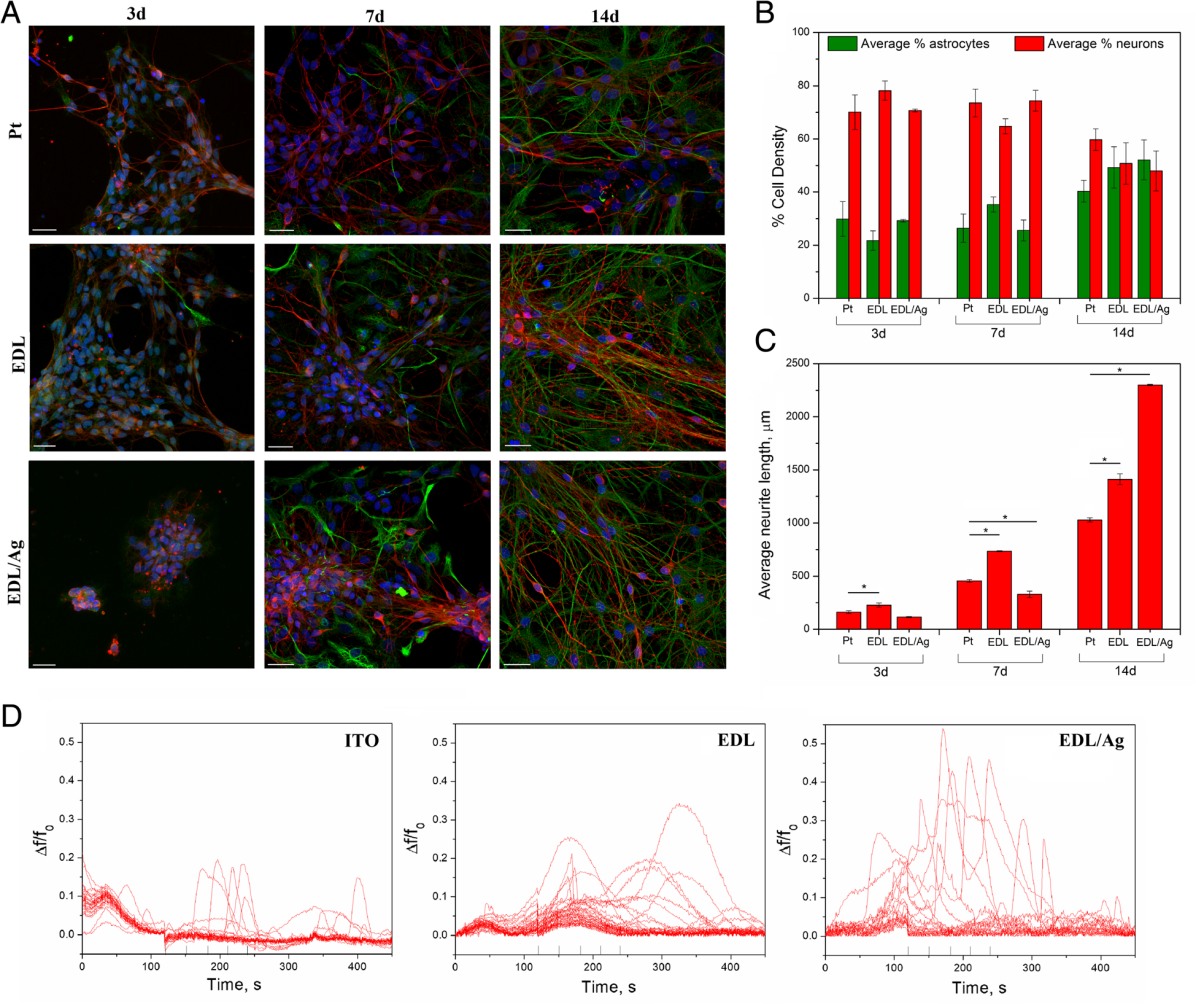
Cytocompatibility of pristine EDL and EDL:Ag nanocomposites. Fluorescent images of primary ventral mesencephalic mixed cell population cultured for 3, 7 and 14 days on each of the experimental and control groups: bare Pt, pristine EDL and EDL:Ag 10:1 composite; neurons are visualized by anti *β*-tubulin III (red), astrocyte cells by anti-GFAP (green) and nuclei by DAPI (blue); the scale bar represents 20 μm (**a**). Cell density (%) analysis of astrocyte and neuron presence on each of the experimental and control groups (**b**) and neural length analysis of the experimental and control groups (**c**); Calcium imaging and substrate stimulation for a bare, EDL-coated and EDL:Ag 10:1-coated ITO electrode: fluorescence tracings show the appearance of Ca^2+^ episodes; short lines highlight the stimulation pulses (**d**);  = *p* < 0.05, *N* = 3

To assess the cytocompatibility of the nanocomposite coatings in vitro, a mixed neural population obtained from the mesencephalon of embryonic Sprague–Dawley rats was cultured on EDL:Ag 10:1 as well as on pristine EDL and magnetron sputter deposited Pt over a period of 3, 7 and 14 days (Fig. 7a). Image analysis of astrocytes and neurons obtained from fluorescent microscopy images revealed a similar trend of astrocyte-to-neuron ratio for all investigated surfaces at individual time points (Fig. 7b). The presence of increased neurons with respect to astrocytes as noted at the initial time point (day 3), gradually decreased to a ratio of approximately 1:1 by the last experimental time point (day 14). The analysis of neurite length on experimental and control substrates (Fig. 7c) showed that after 14 days an average length of 2300 ± 6 μm was observed on EDL:Ag 10:1 formulation, significantly higher than for either pristine EDL (1411 ± 52 μm) or bare Pt (1030 ± 19 μm) electrodes.

For the cell stimulation studies, primary cultures of VM cells were grown on bare, EDL-coated and EDL:Ag 10:1 coated transparent ITO electrodes (to aid imaging) for 18 days and stimulated with a monophasic, negative voltage pulse (− 2 V) for a duration of 500 ms. The recorded traces of changes in background subtracted Fluo4 fluorescence intensity were acquired and shown in Fig. 7d. It could be observed that the highest Δf/f_0_ ratio (equal to 0.5) was achieved on the surface of EDL:Ag 10:1 composite materials, indicating the ability of this nanocomposite coating to increase the stimulation efficiency when compared with both pristine ITO (Δf/f_0_ ratio of 0.2) and pristine EDL-coated ITO (Δf/f_0_ ratio of 0.3).

## Discussion

In this study, the poly(ε-decalactone) (EDL) was synthesized via a ring-opening polymerization process in the presence of a Bi catalyst. Considering the available metal catalysts, bismuth salts or complexes, such as Ph_3_Bi, are notable for their low toxicity [32]. In this context, several studies [33–35] have demonstrated that bismuth compounds belong to the group of least toxic heavy metal compounds, while Bi^3+^ performed even better than Zn^2+^. Moreover, it was found that Ph_3_Bi catalyzes the formation of high molecular masses as demonstrated in [8, 36, 37], which may be a limitation of other catalysts. The viscous nature of EDL was found to facilitate simple coating processes such as dip and spin-coating. Consequently, an electrically conducting composite material was formed with a facile method involving simple mixing of pristine EDL an ethanol dispersion of silver nanowires (AgNWs).

The results of rheological testing showed that although the addition of AgNWs was observed to decrease the critical strain of nanocomposite materials, the observed change in viscoelastic properties was modest, yet sufficient to impart increased material resilience relative to pristine EDL formulations. However, when the ratio of EDL:Ag was decreased to 10:1, the storage modulus as well as gelation frequency was also observed to decrease. This phenomenon was attributed to the agglomeration of AgNWs after some threshold concentration as described previously [38]. The lowest critical strain rate was observed in the composite formulation with an EDL to Ag ratio of 10:1. This phenomena was attributed to a higher concentration of AgNWs reducing polymer entanglement and leading to polymer chain breakages with high strain rates.

While the FTIR spectra confirmed the presence of substantial amount of EDL in all composite formulations, a 10% *w*/*v* solution of EDL in THF was found to give the most homogeneous coating with the uniform thickness. SEM images of a representative EDL:Ag 10:1 composite showed that AgNWs were able to form an extended network within the EDL matrix. It could be observed that a high degree of dispersion was achieved with no apparent bundles of AgNWs, even though the mass ratio of polymer to AgNW utilised here was higher than that described in previous studies [39], confirming that the spin-coating procedure was suitable for producing thin-films of EDL:Ag nanocomposites possessing a uniform distribution of AgNWs. Due to their small diameter (30 nm), large length (100–200 μm) and corresponding high aspect ratio (approx. 5000), individual AgNWs were not observed as straight and rigid wires, but rather as coiled and interconnected woven structures. This type of a metal framework offers advantageous chemical, mechanical and electrical anisotropy which has been shown to provide beneficial interactions with numerous cell types [40].

Materials used for neural interfaces should exhibit suitable electrochemical behaviour and in particular, a tailored charge storage capacity (CSC). High CSC describes the capability of a material to store electric charge, which results in higher charge delivery during electrical stimulation [41]. The CSC depends on both the chemical composition of the material and its physical morphology, and can be increased as a function of the electrode surface area [16]. Consequently, a large electroactive surface area leads to the development of the solid/liquid interface enabling more charge to be trapped within a electrical double layer [41, 42]. Another way to increase CSC is by designing an electrode coating which undergoes a redox reaction, resulting in faradaic charge transfer in the processes of oxidation and reduction [41]. With respect to EDL:Ag composites, it was observed that both of these processes took place. The faradaic process was observed through the presence of a characteristic Ag/Ag^+^ redox couple [43], and the increasing mass ratio of AgNWs in the composite resulted in the expansion of the overall area under the CV curve, leading to the increase in CSC. The CSC of EDL:Ag 10:1, determined in this study, was almost 20 times higher than that of bare Pt substrates and 7 times higher than that of pristine EDL coated Pt electrodes, and was also higher than that for other previously reported biomaterial systems containing nanowires [44], indicating the high efficiency of EDL:Ag 10:1 in storing electric charge, and providing higher charges during electrical stimulation. It was hypothesised that the observed difference in CSC for bare Pt and pristine EDL coated electrodes indicated that pristine polymer is able to accumulate some portion of charge transferred through the electrode, due to its weak capacitive properties.

As well as exhibiting a high charge capacitance expressed in CSC, effective neural interfaces should be also able to inject charge in a stable and controlled manner. It was again noted that EDL:Ag 10:1 coated electrodes possess the highest CIC, greater than the CIC observed with both pristine EDL coated and bare Pt electrodes. Furthermore, among all investigated materials EDL:Ag 10:1 provided the most stable and controlled amount of injected charge, and the high CIC allows for the use of low electrical potentials without compromising the therapeutic effects of stimulation [45]. A comparison of the CIC values with respect to the AgNWs content in EDL:Ag composites indicated that lower AgNWs contents reduced the CIC, a phenomenon previously shown to be due to the lack of a robust percolation network [46], preventing the effective release of stored charge.

To promote both neural stimulation and recording an ideal neural interface should possess advantageous charge transfer characteristics coupled with a low electrochemical impedance profile. Since the majority of protocols describe electrical stimulation with the frequencies between 50 and 300 Hz [30, 31], it can be inferred that EDL:Ag 10:1 coating will outperform bare Pt in both neural stimulation and recording applications. The EDL:Ag 10:1 formulation was also found to have the lowest impedance at 1 kHz, which is used as a benchmark when analysing the efficacy of neural interfaces [47]. The explanation of this phenomenon can be provided by an analysis of the phase angle vs. frequency plot. The observed shift in the frequency peak indicating the distinctive capacitive behaviour of the uncoated and coated electrodes [48] has origins in the mechanisms of charge transfer based on either the double layer effects (pristine EDL) or on faradaic reactions (bare Pt). Upon the addition of AgNWs, the phase angle profile of EDL was changed and the new “faradaic” peak appeared. Furthermore, when the mass ratio of AgNWs to EDL was increased, the relative intensity of the signal changed. Finally, the “double layer” peak disappeareds leaving only a “faradaic” signature, confirming that the capacitive behaviour of EDL:Ag 10:1 is governed mainly by an Ag/Ag^+^ redox reaction.

The results of the in vitro cytocompatibility assessment showed a normal equilibrium between neurons and astrocytes on all types of investigated surfaces. Conversely, major differences were observed with respect to the analysis of neurite length on experimental and control substrates. The EDL:Ag 10:1 formulation was shown to greatly support the outgrowth of neurites in vitro, which after 14 days possessed an average length of 2300 ± 6 μm, 60% longer than those observed in neurons cultured on pristine EDL and 120% longer than those of neurons cultured on bare Pt electrodes. It is worth noting that for all time points pristine EDL was observed to outperform bare Pt with respect to inducing an increase in neurite length. The cell stimulation studies showed the ability of EDL-based coatings to transfer charges suitable for cellular depolarization, through the substantial increase in the Δf/f_0_ ratio when compared with both uncoated and pristine EDL-coated electrodes. This is supposed to be the effect of the unique surface morphology and capacitive properties of the EDL:Ag 10:1 formulation, indicating its efficacy in acting as a robust neural interface material.

## Conclusions

In this study, the applicability of a poly(ε-decalactone)/silver nanowire composite as a neural interface biomaterial was assessed in vitro. When nanocomposites were prepared with polymer-to-nanowire mass ratio of 10:1, AgNWs formed an extended uniformly distributed network within the EDL matrix, resulting in advantageous electrochemical properties. EDL:Ag was shown to possess a significantly increased CSC (8.7 ± 1.0 mC/cm^2^), CIC (84.3 ± 1.4 μC/cm^2^) and low impedance at 1 kHz (194 ± 28 Ω), outperforming both pristine EDL and bare Pt electrodes. Furthermore, a 10:1 EDL:Ag nanocomposite was also observed to support the growth of neurons and promote the outgrowth of neurites, which after 14 days were 60 and 120% longer relative to pristine EDL and bare Pt substrates respectively. Consequently, EDL:Ag nanocomposites were shown to serve as robust neural interface materials, possessing favourable electrochemical characteristics together with high neural cytocompatibility,

## Methods

### Synthesis and characterization of poly(ε-decalactone)

Poly(ε-decalactone) (EDL) was synthesized in bulk by a one-pot-one-step ring-opening polymerization process. The synthesis reaction was conducted in a flask immersed in a controlled temperature oil bath (130 °C). The flask, in which a predetermined amount of ε-decalactone (> 99%, Sigma Aldrich) was added, was purged for 30 min with a nitrogen stream under the surface of the melt. The catalyst (Ph_3_Bi, Gelest) was then added (at 100:1 monomer/catalyst molar ratio) and the magnetic stirrer maintained at 100 rpm. No initiator compound was added to the reaction mixture so the catalyst was activated by the alcohol groups (R-OH) provided by the monomers (H_2_O and impurities). The polymerization was carried out over 7 days. After the corresponding period of reaction time, the product was dissolved in chloroform and precipitated, pouring the polymer solution into an excess of methanol to remove the catalyst impurities and those monomers that had not reacted. Finally, the product was dried at room temperature and then heated at 140 °C for 1 h to ensure the complete elimination of any remaining solvent.

The molecular weight of the homopolymer was determined by gel permeation chromatography using a Waters 1515 GPC device equipped with two Styragel columns (10^2^–10^4^ Ǻ). Chloroform was used as eluent at a flow rate of 1 mL min^− 1^ and polystyrene standards (Shodex Standards, SM-105) were used to obtain a primary calibration curve. The samples were prepared at a concentration of 10 mg in 1.5 mL. Differential scanning calorimetry was performed in a Q200 instrument (TA Instruments). Samples of 6–9 mg were heated from − 85 to 140 °C at 20 °C min^− 1^ to determine the thermal transitions of the polymer.

### Fabrication of EDL: Ag composites

EDL was dissolved in THF (Sigma Aldrich) to form a 10% *w*/*v* solution which was further mixed with a relevant volume of the silver nanowires (AgNWs) dispersed in ethanol (ACS Material, average diameter 30 nm, average length 100–200 μm, silver purity ~ 99.5%, 20 mg/ml).

Four compositions were formed, comprising EDL to AgNWs mass ratio of 100:1, 50:1, 20:1 and 10:1. A glass substrate (25 × 25 × 1.0 mm) was used as the support for the EDL:Ag coating. Prior to the deposition of a composite, glass slides were sputter-coated (Emitech K650XT Sputter Coater, 25 mA, 1 **×** 10^− 3^ mbar, 180 s) with a thin layer of Pt and pre-treated with an oxygen plasma process (Zepto LF, Diener Electronics) for 10 min. Pristine EDL and EDL:Ag coatings were deposited through spin coating (Laurell Technologies Spin Coater) of 0.1 ml of relevant dispersion for 20 s with a speed of 3000 rpm.

### Rheological testing

Dynamical rheological testing was carried out on an Anton Paar MCR 302 via parallel plate geometry to evaluate the viscoelastic properties of different material types. Experiments were carried out using (a) a frequency ramp from 0.01 to 100 Hz under constant 1% strain rate and (b) a strain rate ramp from 0.1 to 100% under constant 1 Hz frequency. In all experiments, the temperature was constant at 37 °C and the distance between the two parallel plates was constant at 0.2 mm.

### Chemical and morphological characterization of EDL:Ag composites

A Varian 660-IR FT-IR Spectrometer was used to collect IR spectra in a range between 3500 and 600 cm^− 1^ for 32 scans. Scanning Electron Microscopy (SEM) images were collected with a Hitachi S-4700 Scanning Electron Microscope operating at 15 kV.

### Electrochemical characterization

Electrochemical studies were performed according to our previously established protocol [49] by means of a PARSTAT 2273 potentiostat in a three-electrode set-up, comprising Pt foil (0.1 mm thickness, 99.9% purity, produced by Mennica-Warsaw, Poland), EDL- and EDL:Ag-coated Pt/glass slide as a working electrode, Ag/AgCl as a reference electrode and glassy carbon rod as an auxiliary electrode. Cyclic voltammograms (CVs) were collected in 0.1 M KCl solution, within the potential range from − 0.8 to 1.0 V (vs. Ag/AgCl) at 100 mV s^− 1^ for 5 CV cycles. The charge storage capacity (CSC) of materials was determined basing on the CV curves according to the formula [50]:

$$ CSC={\int}_{t_1}^{t_2}I(t) dt $$

where *t*_*1*_ is the beginning of CV cycle, *t*_*2*_ is the end of CV cycle, and *I* is the current.

Electrochemical impedance spectra were collected in a 0.1 M KCl solution with frequencies ranging from 100 to 100 kHz, an AC amplitude of 40 mV (vs. Ag/AgCl) and a DC potential equal to 0 V (vs. Ag/AgCl).

The chronoamperometric curves representing a physiologically relevant [26–31] single biphasic potential pulse consisting of a 5 ms application of a reduction potential followed by a 5 ms application of an oxidative potential were used to determine the charge injection capacity (CIC) of the materials.

### In vitro biological characterization

Cytocompatibility of the pristine EDL and EDL:Ag composites was determined with primary cultures of a mixed neural population obtained from the ventral mesencephalon of E14 rat embryos [49]. The embryos were obtained by laparotomy from time-mated female Sprague-Dawley rats delivered by Charles River Laboratories. After arrival and 1 day accommodation period, the rats were decapitated under terminal anaesthesia induced by the inhalation of isoflurane [17, 51]. Neurons and astrocyte cell populations were visualized through the indirect double-immunofluorescent labelling [17, 19], with the use of an Olympus Fluoview 1000 Confocal Microscope (scan size of 1024 × 1024 at a ratio 1:1 and 60× magnification). To analyse cell density, the number of nuclei corresponding to neurons and astrocytes were counted in an area of 211.97 μm × 211.97 μm in at least 20 random images taken from test and control groups [19]. To quantify the average neurite length, the stereological methods was used [52] and the following formula [53]:

$$ L= nT\frac{\pi }{2} $$

where: *L* is neurite length (μm), *n* is the number of times neurites intersect with grid lines, *T* is distance between grid lines (μm).

For all experimental and control groups, the biological experiments included three biological replicates. The results were expressed as the mean of the values ± standard error of the mean, and the statistical significance was determined through a t-test (*p* < 0.05).

Stimulation of pristine EDL and EDL:Ag 10:1 films deposited on transparent ITO/PET foil (Sigma Aldrich, surface resistivity 60 Ω/sq) was performed with a primary culture of VM cells cultured on the substrates for 18 days. The changes in intracellular calcium were measured as described previously [19, 51]. Electrical stimulation was achieved by the delivery of a monophasic, negative voltage pulse (− 2 V) for 500 ms (1 pulse per 30 s), using a software-controlled constant voltage stimulator connected to the chamber via Pt contacts. The chamber was mounted on the stage of a Zeiss Axiovert 200 inverted microscope equipped with a 10 position Orbit I filterwheel (Improvision), for excitation (488 nm) and emission (510 nm low-pass filter). The emission light was collected every second with an Orca 285 camera (Hamamatsu). The changes in Ca^2+^ were expressed as Δf/f_0_, where f_0_ denotes the baseline and Δf denotes the rise over the baseline.

## Abbreviations

AgNWs: Silver nanowires
CIC: Charge injection capacity
CSC: Charge storage capacity
CV: Cyclic voltammetric
EDL: Poly(ε-decalactone)
EDL:Ag: Poly(ε-decalactone)/silver nanowire
FTIR: Fourier-transform infrared spectroscopy
GFAP: Glial fibrillary acidic protein
ITO: Indium tin oxide
SEM: Scanning electron microscopy
εDL: ε-decalactone
